# Risk of Gestational Diabetes Mellitus in relation to Plasma Concentrations of Fatty Acid-Binding Protein 4: A Nested Case-Control Study in China

**DOI:** 10.1155/2021/6681432

**Published:** 2021-07-28

**Authors:** Chuyao Jin, Lizi Lin, Na Han, Zhiling Zhao, Xiangrong Xu, Shusheng Luo, Jue Liu, Haijun Wang

**Affiliations:** ^1^Department of Maternal and Child Health, School of Public Health, Peking University, Beijing, China; ^2^Tongzhou Maternal and Child Health Hospital, Beijing, China; ^3^Department of Epidemiology and Biostatistics, School of Public Health, Peking University, Beijing, China

## Abstract

**Objective:**

The study is aimed at examining the effects of fatty acid-binding protein 4 (FABP4) on insulin resistance and gestational diabetes mellitus (GDM).

**Methods:**

Based on a prospective birth cohort in Beijing, China, we conducted a nested case-control study and analyzed 135 GDM case-control pairs matched by age and the gestational week when they took the oral glucose tolerance test. We performed linear regression to analyze the association of plasma FABP4 concentrations with insulin resistance. We used logistic regression to estimate odds ratios (ORs) of FABP4 for GDM, controlling for potential confounders, including dietary intake and physical activity.

**Results:**

Plasma FABP4 levels in the first and second trimesters were positively associated with fasting insulin and homeostasis model assessment for insulin resistance (HOMA-IR) in the second trimester (both *P* < 0.001). Compared with those in the lowest FABP4 tertile, women in the highest tertile of FABP4 levels in the first and second trimesters had 1.053 times (OR = 2.053, 95% CI 1.091 to 3.863) and 1.447 times (OR = 2.447, 95% CI 1.305 to 4.588) higher risk of developing GDM.

**Conclusions:**

Elevated FABP4 levels in the first and second trimesters were associated with a higher level of insulin resistance and greater GDM risk, indicating FABP4 might predict women with high risk of developing GDM.

## 1. Introduction

Gestational diabetes mellitus (GDM) is an increasing public health concern, affecting 2% to 25% of pregnancies worldwide [1]. It was estimated in 2019 that globally 17.1 million women with live births had GDM in pregnancy [2]. GDM causes significant health risks for the mothers and offspring, resulting in a considerable economic burden at both societal and individual levels [3, 4]. However, GDM is generally diagnosed in the later phase of pregnancy, when preexisting metabolic dysfunction might have affected the mother and the fetus, and only a short intervention window is left. Thus, it is beneficial to find an appropriate biomarker in early pregnancy to detect women with high GDM risk to improve their health outcomes and that of their children.

Fatty acid-binding proteins (FABPs) can reversibly bind long-chain fatty acids and regulate lipid trafficking and responses in cells [5, 6]. Each FABP regulates metabolic and inflammatory signaling pathways uniquely [7]. Among the FABPs, fatty acid-binding protein 4 (FABP4) is mainly expressed in adipocytes and macrophages and plays a vital role in the pathogenesis of insulin resistance and type 2 diabetes [8, 9], indicating that it might also have predictive value for GDM. However, previous studies mainly compared FABP4 concentrations between GDM cases and controls at or after the diagnosis of GDM [10–16]. Only four studies analyzed the levels of FABP4 in the first trimester and yielded inconsistent results [17–20]. They were either limited by not adjusting for some important lifestyle factors associated with GDM, such as dietary intake and physical activity [17, 19, 20], or measuring the concentrations of FABP4 only once, unable to depict the dynamic effect of FABP4 on GDM [18, 19].

Thus, in the current study, a nested case-control design will be used to prospectively investigate the associations of FABP4 levels with insulin resistance and GDM after accounting for lifestyle factors. Since FABP4 levels may change as pregnancy progresses, we analyzed these associations both in the first and second trimesters.

## 2. Methods

### 2.1. Study Population

This study was nested within the Peking University Birth Cohort in Tongzhou (PKUBC-T), which is aimed at evaluating the associations of prepregnant and prenatal exposure with the health outcomes of mothers and their offspring. From June 2018, eligible pregnant women aged 18 to 45 were enrolled successively before 14 weeks of gestation at Tongzhou Maternal and Child Health Hospital. Exclusion criteria included women with pregestational diabetes mellitus, cardiovascular diseases, kidney diseases, liver diseases, and autoimmune diseases. Pregnant women routinely underwent the oral glucose tolerance test (OGTT) during their second trimester. By February 2019, 3304 women enrolled in the PKUBC-T took the OGTT; 593 (17.9%) of which were diagnosed with GDM. We further excluded women with a family history of diabetes, GDM history, thyroid diseases, polycystic ovary syndrome, alcohol consumption, and cigarette smoking. Then, we randomly selected 135 women with GDM to form the case group. Non-GDM controls were also randomly chosen and matched for every GDM case by age (±2 years) and the gestational week at taking OGTT. All subjects gave informed consent, and this study was approved by the institutional review boards at Peking University (IRB00001052-18003).

### 2.2. Data Collection

At baseline, the information of participants was collected from self-administered questionnaires and hospital information system. Trained nurses measured and recorded participants' heights, weights, and blood pressures. Prepregnancy body mass index (BMI) was calculated as self-reported prepregnancy weight divided by the square of measured height (kg/m^2^). Gestational weight gain (GWG) before OGTT was calculated as weight measured within 2 weeks before taking OGTT minus prepregnancy weight. Physical activity was estimated by the short form of the International Physical Activity Questionnaire [21] and modelled as a continuous variable (MET-min/week). Dietary intake was evaluated by daily calorie intake, which was calculated according to two 24 h recalls on nonconsecutive days [22].

### 2.3. Laboratory Measurements

Blood samples were collected at enrollment (<14 weeks) and in the second trimester (25-28 weeks). Gestational week at each blood collection was calculated based on the date of the last menstrual period, which was confirmed by ultrasound measurement. Fasting plasma glucose and serum lipid profile, including triglyceride, high-density lipoprotein (HDL) cholesterol, alanine transaminase (ALT), and aspartate aminotransferase (AST), were analyzed by standard detection methods. The concentrations of fasting plasma insulin and FABP4 were batch measured by sandwich ELISA kits (R&D Systems China, Shanghai) in duplicate according to the manufacturers' instructions. Assessment of samples was blinded to case/control status.

### 2.4. Outcome Assessment

The main outcomes were insulin resistance and GDM. Insulin resistance was assessed by the homeostasis model assessment of insulin resistance index (HOMA-IR) using the equation as follows: HOMA‐IR = fasting glucose (mmol/L) × fasting insulin (*μ*U/mL)/22.5 [23]. GDM was diagnosed according to the standard set by the International Association of Diabetes and Pregnancy Study Groups [24]. All subjects took a 75 g OGTT in the morning after fasting for at least 8 hours. GDM was diagnosed if there was at least one abnormal value for fasting glucose (≥5.1 mmol/L), 1 h glucose (≥10.0 mmol/L), or 2 h glucose (≥8.5 mmol/L).

### 2.5. Statistical Analyses

Continuous variables were presented as means ± SDs or medians (interquartile ranges (IQRs)) depending on their distributions. Differences in characteristics between GDM cases and controls were evaluated by Student's *t*-test (normal distribution) or Wilcoxon rank-sum test (nonnormal distribution) for continuous variables and chi-square test for categorical variables. Changes in FABP4 from the first trimester to the second trimester were assessed by the Wilcoxon signed-rank test. We conducted multivariable linear regression to analyze the effects of FABP4 on fasting insulin and HOMA-IR. FABP4 levels were then categorized into tertiles, and the lowest category was the reference group. We performed logistic regression to estimate odds ratios (ORs) and 95% confidence intervals (CIs) of FABP4 tertiles for GDM, adjusting for potential confounders including maternal age, gestational week at enrollment, and education in model 1 and further adjusting for prepregnancy BMI, GWG before OGTT, physical activity, daily calorie intake, SBP, DBP, triglyceride, HDL cholesterol, ALT, and AST in model 2. Linear trends were tested by entering the median value of each category of FABP4 tertile in the logistic regression model as a continuous variable. We also calculated ORs and 95% CIs of GDM associated with 1 log increment in the FABP4 levels. We used SAS 9.4 for statistical analyses. A *P* value less than 0.05 was considered statistically significant.

## 3. Results

**Figure 1:**
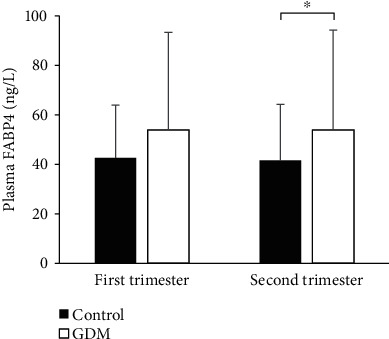
Concentrations of plasma FABP4 levels in GDM cases and controls. ^†^GDM cases vs. controls in the first trimester (median (interquartile range)): 53.3 (33.1~93.2) ng/L vs. 42.4 (32.6~63.8) ng/L, *P* = 0.068. ^‡^GDM cases vs. controls in the second trimester (median (interquartile range)): 53.8 (36.8~94.1) ng/L vs. 41.6 (33.4~64.1) ng/L, *P* = 0.008. ^∗^*P* < 0.05. Abbreviations: GDM: gestational diabetes mellitus; FABP4: fatty acid-binding protein 4.

Baseline characteristics of subjects are shown in Table 1. We found no differences in maternal age, gestational week at enrollment, parity, education, prepregnancy BMI, GWG before OGTT, physical activity, dietary intake, SBP, DBP, triglyceride, HDL cholesterol, ALT, and AST between two groups. GDM cases had significantly higher levels of fasting glucose, fasting insulin, and HOMA-IR in the first trimester. As shown in Figure 1, FABP4 concentrations of GDM cases and controls were 53.3 (33.1~93.2) ng/L vs. 42.4 (32.6~63.8) ng/L (*P* = 0.068) in the first trimester and 53.8 (36.8~94.1) ng/L vs. 41.6 (33.4~64.1) ng/L (*P* < 0.05) in the second trimester, respectively. From the first trimester to the second trimester, the concentrations of FABP4 remained similar in both groups (*P* > 0.05).

As shown in Table 2, FABP4 levels in the first and second trimesters were positively associated with fasting insulin and HOMA-IR in the second trimester, after controlling for maternal age, gestational week at enrollment, and education (all *P* < 0.001). Further adjusting for prepregnancy BMI, GWG before OGTT, physical activity, dietary intake, SBP, DBP, triglyceride, HDL cholesterol, ALT, and AST did not attenuate this association.

We then categorized FABP4 levels into tertiles. As shown in Table 3, the highest tertile had a higher proportion of GDM cases compared with the lowest tertile (60.0% vs. 47.8% in the first trimester; 57.8% vs. 38.9% in the second trimester). In the fully adjusted model, positive associations of FABP4 concentrations in the first trimester (highest tertile: OR = 2.053, 95% CI 1.091 to 3.863; *P* for trend = 0.008) and the second trimester (highest tertile: OR = 2.447, 95% CI 1.305 to 4.588; *P* for trend = 0.015) with GDM risk were observed. The adjusted OR for GDM with per 1 log increment of FABP4 concentrations was 2.895 (95% CI 1.157 to 7.249) in the first trimester and was 3.575 (95% CI 1.343 to 9.517) in the second trimester.

## 4. Discussion

In the nested case-control study, we found that increased FABP4 concentrations in the first and second trimesters were associated with a greater risk of developing GDM. We further provided evidence that higher FABP4 concentrations early in pregnancy may indicate subsequent higher insulin resistance levels. Additional adjustment for potential confounders, including dietary intake and physical activity, did not alter these results. The current study provides novel insights into the pathogenesis of GDM.

Previous studies regarding the associations of FABP4 with the risk of GDM mainly focused on the levels of FABP4 at or after the diagnosis of GDM and showed women with GDM had relatively higher FABP4 concentrations than healthy controls in late pregnancy [10–16]. Only four prospective studies measured FABP4 levels in the first trimester [17–20]. Sharafeldeen et al. [19] showed that FABP4 concentrations at 6 weeks of gestation were positively related to the development of GDM at 24 weeks of gestation. Tu et al. [18] found that higher FABP4 levels in the first trimester were correlated with greater GDM risk with associated adjusted OR of 3.57 (95% CI 1.99 to 6.11) for the highest quartile. In another study by Francis et al. [20], FABP4 levels in 10–14 gestational weeks and 15–26 gestational weeks were positively associated with increased risk of developing GDM, after adjustment for maternal age, family history of diabetes, gestational week of blood collection, parity, and prepregnancy BMI. Inconsistent with the aforementioned studies, Guelfi et al. [17] did not find significant differences in FABP4 concentrations between cases and controls at 14 and 28 weeks of pregnancy. On the one hand, the subjects in this study were from a previously conducted randomized controlled trial and were restricted to women with GDM history. Thus, it is not a population-based observational study, and these women may have distinctive metabolic profiles. On the other hand, differences in race/ethnicity and diagnostic criteria for GDM among these studies may contribute to divergent findings. With the strength of analyzing FABP4 concentrations at two time points in early pregnancy and adjusting for important lifestyle factors, including dietary intake and physical activity, our study identified that the concentration of FABP4 in the first trimester could predict GDM risk and its predictive ability was consistent from the first trimester to the second trimester.

In our study, we did not find significant increases in FABP4 concentrations from the first trimester to the second trimester in both case and control groups, which was consistent with the following two studies. Guelfi et al. [17] found that FABP4 concentrations remained similar at 14 and 28 gestational weeks. In the study by Francis et al. [20], no significant differences between cases and controls were found of changes in FABP4 levels from 10–14 gestational weeks to 15–26 gestational weeks. The possible explanation is that the progressive increase in insulin resistance mainly happened in the third trimester until delivery, not in the early pregnancy [3]. Thus, noticeable changes in FABP4 concentrations may also emerge later. As shown in a study by Zhang et al. [14], from the second trimester to the third trimester, FABP4 levels increased significantly among GDM cases. The stable concentrations of FABP4 from the first trimester to the second trimester might be helpful for predicting women with high GDM risk in early pregnancy.

We further found that FABP4 levels in the first trimester and the second trimester were positively correlated with insulin resistance in the second trimester. Similarly, previous studies also observed the positive association between FABP4 levels at 6, 24-28, or 23-30 weeks of gestation and insulin resistance [12, 13, 19]. This finding is biologically plausible. FABP4 regulates lipid trafficking and responses at the cellular level [5, 6]. Elevated FABP4 concentrations may impair the ability of adipocytes to take up and retain free fatty acids, resulting in ectopic lipid accumulation, a critical contributing factor to insulin resistance, type 2 diabetes, and GDM [25]. Previous studies showed that FABP4 inhibition reduced triacylglycerol content in human trophoblasts [26]. Likewise, studies in animal models found that, even being challenged by high-fat diets, FABP4-deficient mice were protected from hyperglycemia, hyperinsulinemia, and insulin resistance [8, 27]. Based on the existing evidence, the reduction in FABP4 levels may improve insulin sensitivity and lower diabetes risk.

The study has some limitations. First, the sample size is limited for subgroup analyses. Hence, we could not estimate if FABP4 interacts with other factors such as prepregnancy BMI on the development of GDM. Second, the sample only consisted of Chinese women. Thus, the results may not be generalized to other races/ethnicities.

## 5. Conclusions

FABP4 levels in the first and second trimesters were associated with higher levels of insulin resistance and greater risk of GDM, after controlling for dietary intake, physical activity, and other possible confounders. The findings suggested the promising role of FABP4 in early pregnancy to predict women with high GDM risk. Further studies need to validate these findings and investigate the feasibility of conducting specific nutritional or medical interventions on the concentrations of FABP4 to improve insulin sensitivity and reduce GDM risk during early pregnancy.

## Figures and Tables

**Table 1:** Baseline characteristics of subjects in the first trimester.

	GDM cases (*N* = 135)	Controls (*N* = 135)	*P* value
Age (years)	29 (28-33)	29 (28-33)	1.00
Gestational week at enrollment	10 (9-12)	10 (9-12)	0.36
Parity			0.90
0	60.0	59.3	
≥1	40.0	40.7	
Education > 12 years (%)	81.5	79.3	0.55
Prepregnancy BMI (kg/m^2^)	22.2 (20.3-25.1)	22.0 (19.9-24.8)	0.45
GWG before OGTT (kg)	8.6 (6.2-11.0)	8.6 (6.4-10.5)	0.56
Weekly PA time (MET-min/week)	693 (238-1386)	693 (198-1386)	0.78
Daily calorie intake (kcal/d)	1272 (1031-1630)	1242 (936-1683)	0.70
SBP (mmHg)	108 (101-116)	110 (102-119)	0.43
DBP (mmHg)	67 (62-73)	67 (61-72)	0.66
Fasting glucose (mmol/L)	5.0 (4.8-5.0)	4.8 (4.6-5.0)	0.02
Fasting insulin (*μ*U/mL)	45.3 (22.4-68.8)	28.1 (19.8-52.8)	0.02
HOMA-IR	9.8 (5.1-15.1)	5.8 (4.1-11.6)	0.01
Triglyceride (mmol/L)	1.2 (0.9-1.4)	1.0 (0.9-1.3)	0.07
HDL cholesterol (mmol/L)	1.7 (1.4-1.9)	1.8 (1.5-1.9)	0.26
ALT (U/L)	13 (10-23)	12 (10-19)	0.33
AST (U/L)	14 (13-18)	15 (13-17)	0.92

Data were presented as medians (interquartile ranges) or %. Abbreviations: GDM: gestational diabetes mellitus; BMI: body mass index; GWG: gestational weight gain; OGTT: oral glucose tolerance test; PA: physical activity; SBP: systolic blood pressure; DBP: diastolic blood pressure; HDL: high-density lipoprotein; ALT: alanine transaminase; AST: aspartate aminotransferase.

**Table 2:** Multivariable linear regression analysis for the associations of FABP4 with fasting insulin and HOMA-IR.

	Fasting insulin in the second trimester		HOMA-IR in the second trimester	
	*β*	*P* value	*β*	*P* value
FABP4 in the first trimester				
Model 1^†^	0.430	<0.001	0.094	<0.001
Model 2^‡^	0.433	<0.001	0.095	<0.001
FABP4 in the second trimester				
Model 1^†^	0.502	<0.001	0.109	<0.001
Model 2^‡^	0.498	<0.001	0.109	<0.001

^†^Adjusted for maternal age, gestational week at enrollment, and education. ^‡^Further adjusted for prepregnancy body mass index, gestational weight gain before oral glucose tolerance test, physical activity, daily calorie intake, systolic blood pressure, diastolic blood pressure, triglyceride, high-density lipoprotein cholesterol, alanine transaminase, and aspartate aminotransferase. Abbreviation: FABP4: fatty acid-binding protein 4.

**Table 3:** Odds ratios of GDM by FABP4 levels in the first and second trimesters.

	Number of case (%)	Adjusted OR (95% CI)^†^	Adjusted OR (95% CI)^‡^
Tertiles of FABP4 in the first trimester (range, ng/L)			
T1 (<35.8)	43 (47.8)	ref	ref
T2 (35.8~61.6)	38 (42.2)	0.798 (0.442-1.440)	0.866 (0.469-1.596)
T3 (>61.6)	54 (60.0)	1.607 (0.884-2.920)	2.053 (1.091-3.863)
*P* for trend		0.045	0.008
Per 1 log increment		2.272 (0.974-5.452)	2.895 (1.157-7.249)
Tertiles of FABP4 in the second trimester (range, ng/L)			
T1 (<37.9)	35 (38.9)	ref	ref
T2 (37.9~62.0)	48 (53.3)	1.772 (0.974-3.224)	1.970 (1.049-3.698)
T3 (>62.0)	52 (57.8)	2.134 (1.168-3.898)	2.447 (1.305-4.588)
*P* for trend		0.032	0.015
Per 1 log increment		3.084 (1.199-7.930)	3.575 (1.343-9.517)

^†^Adjusted for maternal age, gestational week at enrollment, and education. ^‡^Further adjusted for prepregnancy body mass index, gestational weight gain before oral glucose tolerance test, physical activity, daily calorie intake, systolic blood pressure, diastolic blood pressure, triglyceride, high-density lipoprotein cholesterol, alanine transaminase, and aspartate aminotransferase. Abbreviations: GDM: gestational diabetes mellitus; FABP4: fatty acid-binding protein 4.

## Data Availability

The datasets analyzed during the current study are not publicly available but are available from the corresponding author on reasonable request.
